# Deep learning approaches for noncoding variant prioritization in neurodegenerative diseases

**DOI:** 10.3389/fnagi.2022.1027224

**Published:** 2022-11-18

**Authors:** Alexander Y. Lan, M. Ryan Corces

**Affiliations:** ^1^Gladstone Institute of Neurological Disease, San Francisco, CA, United States; ^2^Gladstone Institute of Data Science and Biotechnology, San Francisco, CA, United States; ^3^Department of Neurology, University of California San Francisco, San Francisco, CA, United States

**Keywords:** genomics, machine learning, neurodegeneration, noncoding genetic variation, gene regulation

## Abstract

Determining how noncoding genetic variants contribute to neurodegenerative dementias is fundamental to understanding disease pathogenesis, improving patient prognostication, and developing new clinical treatments. Next generation sequencing technologies have produced vast amounts of genomic data on cell type-specific transcription factor binding, gene expression, and three-dimensional chromatin interactions, with the promise of providing key insights into the biological mechanisms underlying disease. However, this data is highly complex, making it challenging for researchers to interpret, assimilate, and dissect. To this end, deep learning has emerged as a powerful tool for genome analysis that can capture the intricate patterns and dependencies within these large datasets. In this review, we organize and discuss the many unique model architectures, development philosophies, and interpretation methods that have emerged in the last few years with a focus on using deep learning to predict the impact of genetic variants on disease pathogenesis. We highlight both broadly-applicable genomic deep learning methods that can be fine-tuned to disease-specific contexts as well as existing neurodegenerative disease research, with an emphasis on Alzheimer’s-specific literature. We conclude with an overview of the future of the field at the intersection of neurodegeneration, genomics, and deep learning.

## Introduction

Alzheimer’s disease (AD) is a progressive neurodegenerative disorder and the leading cause of dementia worldwide. Recent models have projected that the number of people with dementia will increase from approximately 57 million people in 2019 to 153 million people by 2050 (GBD Dementia Forecasting Collaborators, 2019). Despite recent improvements in our understanding of AD pathophysiology and the emergence of newly proposed treatment strategies, no effective therapy currently exists to fully prevent or reverse the effects of AD (Long and Holtzman, 2019). Given that genetic factors account for an estimated 60–80% of an individual’s risk of developing AD (Sims et al., 2020), an improved understanding of the genetic architecture of AD and the affected biological pathways in disease pathogenesis can inform better prognostication and drug development.

Since 2007, numerous genome-wide association studies (GWASs) have identified dozens of potential risk loci containing genetic variants, or single-nucleotide polymorphisms (SNPs), that have a high statistical correlation with AD (Andrews et al., 2020). However, the true promise of these GWASs has yet to be realized due to multiple challenges involved in pinpointing the true causal variant, its corresponding gene target, and the cell type(s) affected. Underlying each of these challenges is the observation that nearby genetic variants are often co-inherited, and thus statistically indistinguishable, due to non-random segregation of alleles during meiotic recombination, a phenomenon known as linkage disequilibrium (Schaub et al., 2012). Further exacerbating this challenge is the observation that greater than 90% of all GWAS-implicated variants map to noncoding regions of the genome (Maurano et al., 2012). Unlike variants that affect coding sequences which have predictable downstream amino acid alterations, assigning function to noncoding variants is comparatively less straightforward as we lack a fundamental understanding of how sequence changes affect the activity of noncoding gene regulatory elements (Gallagher and Chen-Plotkin, 2018).

Within the nucleus, gene expression is controlled by an intricate interplay between multiple molecular mechanisms. The chromatin state (the post-translational modification of histone tails that is associated with differing gene regulatory potential), the methylation of cytosine bases (“DNA methylation”), and the binding of sequence-specific transcription factor proteins (the “cis-regulatory code”) all influence a cell’s complex expression profile. In the right epigenetic context, a TF recognizes and binds to a specific DNA sequence called a “motif,” initiating an incompletely understood cascade of events that ultimately leads to a change in gene expression. In this way, genetic variants within the noncoding genome could affect TF binding, thus altering downstream gene expression and linked cellular behaviors. To begin to predict which noncoding variants have plausible effects on regulatory element activity and thus could play a role in disease predisposition, an in depth understanding of the gene regulatory circuitry of the brain is required. To this end, functional genomics techniques that analyze chromatin accessibility, gene expression, three-dimensional (3D) chromatin conformation, histone modifications, and other chromatin features have been employed to characterize cell type- and cell state-specific gene regulatory landscapes in the brain.

Genomics is inherently a data-driven science, and the genome-scale assays utilized for chromatin and transcriptome profiling generate vast amounts of complex data. With the growth of high-throughput sequencing technology, researchers have increasingly turned to machine learning (ML) in order to effectively discover intrinsic relationships and patterns in such large amounts of data (Libbrecht and Noble, 2015). However, the potential of traditional ML algorithms is often limited by the complex task of feature extraction, where researchers manually decide which variables would be most important to use in training the model. Once identified, these features would be extracted through preprocessing of the raw data into new handcrafted representations for training inputs (Storcheus et al., 2019). Not only is this a time-intensive process requiring multiple iterations and careful thought, but subtle changes with feature selection can have substantial effects on model performance.

Deep learning (DL) is a subdiscipline of ML that circumvents this challenge of feature engineering by using multiple layers to capture salient features in the model’s internal weights during training (Goodfellow et al., 2016). In the last decade, DL models have emerged as the state-of-the-art models for complex pattern recognition in high-dimensional data and have been instrumental in fields such as natural language processing, computer vision, and bioinformatics (LeCun et al., 2015).

In this review, we give a brief background of DL, focusing specifically on supervised DL, which encompasses models trained with labeled data (pairs of input and output values such as DNA sequences and associated genomic data). We then highlight recent DL innovations in genomics and neurodegenerative disease research, with a focus on AD as it’s the most studied neurodegenerative disease to date. In particular, we center our discussion on the task of noncoding variant prioritization, the process by which functional variants with the potential to affect an individual’s disease predisposition are isolated from large sets of GWAS variants. DL algorithms are particularly well-suited for this “variant effect prediction” task because they are computational representations of the biological relationship between a DNA sequence and its genomic features. The purpose of this review is to grant readers a complete picture of the current state-of-the-art DL methods that can be used for variant effect prediction and clearly represent the benefits and drawbacks of each given approach. Crucially, the adoption of DL techniques has tremendous potential in neurodegeneration research, where DL computational methods that are currently underutilized could serve as a supplementary analytical platform to traditional genomic techniques and improve our understanding of disease pathogenesis.

## Deep learning

**Figure. 1:**
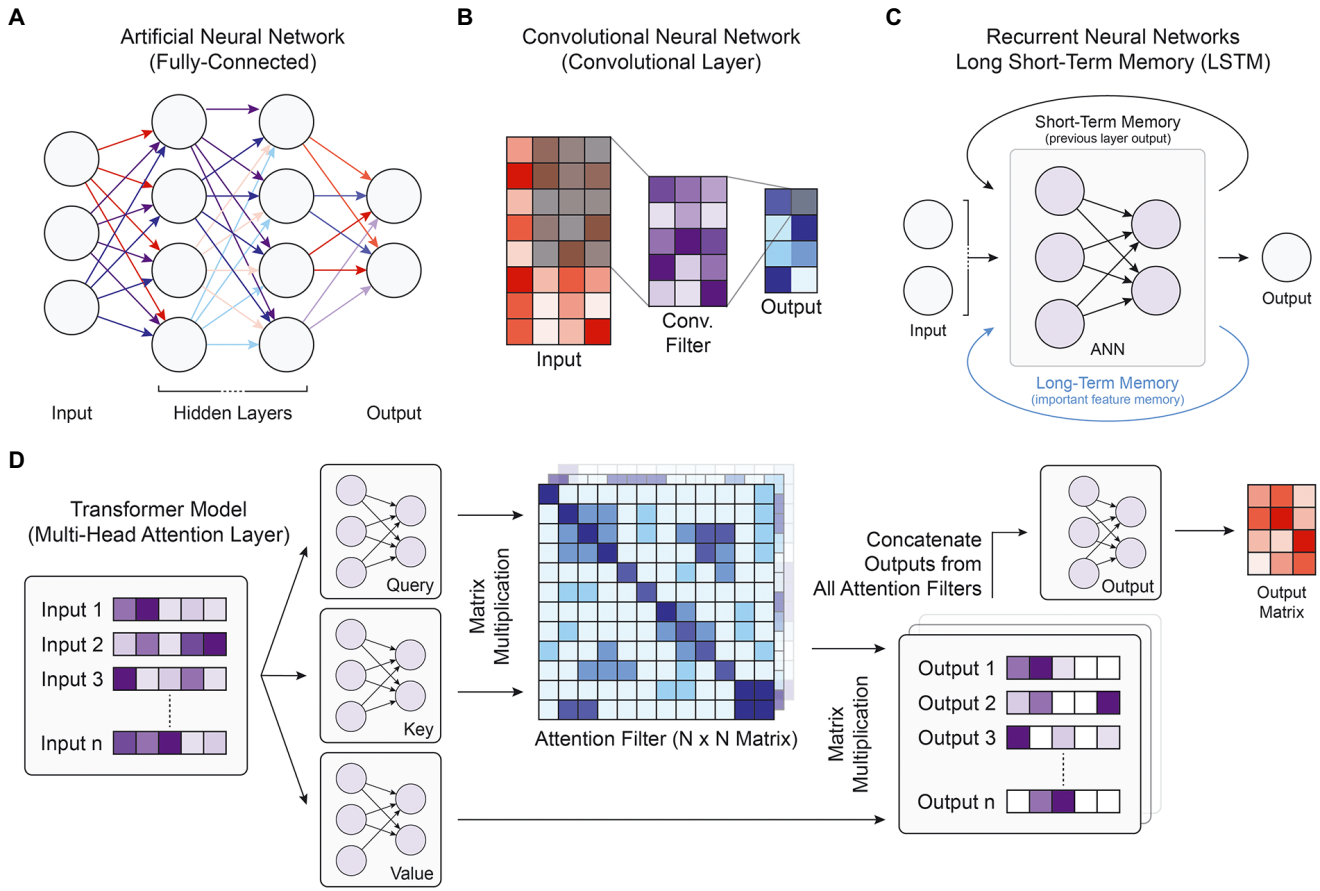
Model and layer architectures. **(A)** Diagram of the fully-connected architecture present in ANNs. Every node is connected with all nodes of the previous layer and all nodes of the following layer. **(B)** Diagram of a single convolutional filter within a single convolutional layer. Every element in the shaded input matrix is multiplied by the corresponding weight in the convolutional filter and combined to form one output value in the shaded output square. (**C)** Depiction of the recurrent neural network architecture, where the primary ANN block takes the current input along with memory information stored over short or long distances. (**D)** Flowchart of the transformer multi-head attention layer, which first takes a list of inputs and passes them through three ANN blocks. Together, the query and key matrix outputs form attention filters, which when multiplied with the outputs of the value matrix, generates a list of filtered output matrices. Each attention filter may highlight a different part of the input. The final output ANN is used to reduce the number of dimensions back to the original input size.

To understand the competing design philosophies behind modern DL model architectures, one must first understand the basic structure of a deep neural network, the foundational construct for most DL models. The fundamental unit of a deep neural network is the artificial neuron. An input vector of real values is passed into the neuron, which calculates the weighted sum of those values and passes it through a nonlinear transformation function (Goodfellow et al., 2016). DL models are constructed of multiple layers of neurons that can receive input data from neurons in the previous layer and output values to neurons in the following layer **(**Figure 1A). The input into the first layer of the deep neural network is generally a matrix of real values, while the output of the last layer is the desired prediction. The intermediate layers are referred to as “hidden layers,” and although generally not human-interpretable, represent the complex nonlinear relationship between the input and output values.

Deep learning frameworks, such as PyTorch (Paszke et al., 2019), Keras on Tensorflow (Abadi et al., 2016), and JAX (Frostig et al., 2018), have been instrumental for the success and proliferation of DL models. These software libraries handle the elementary operations behind model development, including matrix multiplication for forward propagation of an input matrix through the model and backpropagation for updating model weights to better fit the desired relationship. Model training is a critical part of model development and is the process by which weights are adapted to minimize the loss function, which quantifies the difference between the expected outcome and the model’s prediction. During training, the backpropagation algorithm takes the partial derivative of the loss function with respect to each of the weights in the network and iteratively updates the weights to descend the gradient by a factor of the learning rate (Schmidhuber, 2015).

The researcher accounts for the other aspects of model development, namely data preparation and hyperparameter optimization. After task-specific preprocessing methods that can assist with feature extraction and selection, data is partitioned into three distinct sets: the training set, which is used to update model weights; the validation set, which is used during training to check model performance; and the test set, which is used to determine the final model accuracy metrics after training. To assess a model’s performance under conditions it has not previously encountered, the data must be partitioned such that the validation and test sets represent samples the model could not have memorized during training. In genomics, this distinction is traditionally achieved by withholding entire chromosomes or cell types during training. Common metrics used to evaluate model performance on the test set include the Pearson correlation coefficient and Spearman correlation coefficient for regression tasks or the area under the receiver operating characteristic (AUROC) and area under the precision-recall curve (AUPRC) for classification tasks. Hyperparameters, which include the model architecture, learning rate, batch size, and other training variables, are adjusted to maximize model performance while avoiding overfitting, the phenomenon where the model does not generalize well to data points outside the training set. Proper monitoring of the loss function of the training and validation sets during the training process gives insight into the best time to stop training.

### Deep learning architectures

Artificial neural networks (ANNs), also known as multi-layer perceptrons or feed-forward neural networks, consist of fully connected layers where every node (neuron) takes an input from every node in the previous layer of the network, and the overall ANN models the propagation of stimuli across synapses in the brain (Figure 1A; Krogh, 2008). Although ANNs have been used in genomics research to prioritize candidate causal SNPs for disease (Quang et al., 2015), predict enhancer activity (Liu et al., 2016), or predict RNA splicing (Xiong et al., 2015), convolutional neural networks (CNNs), recurrent neural networks, and transformer models integrate the fully connected layer into more advanced structures that have been able to better capture biological patterns in the genomic data and achieve higher performance metrics.

**Figure 2:**
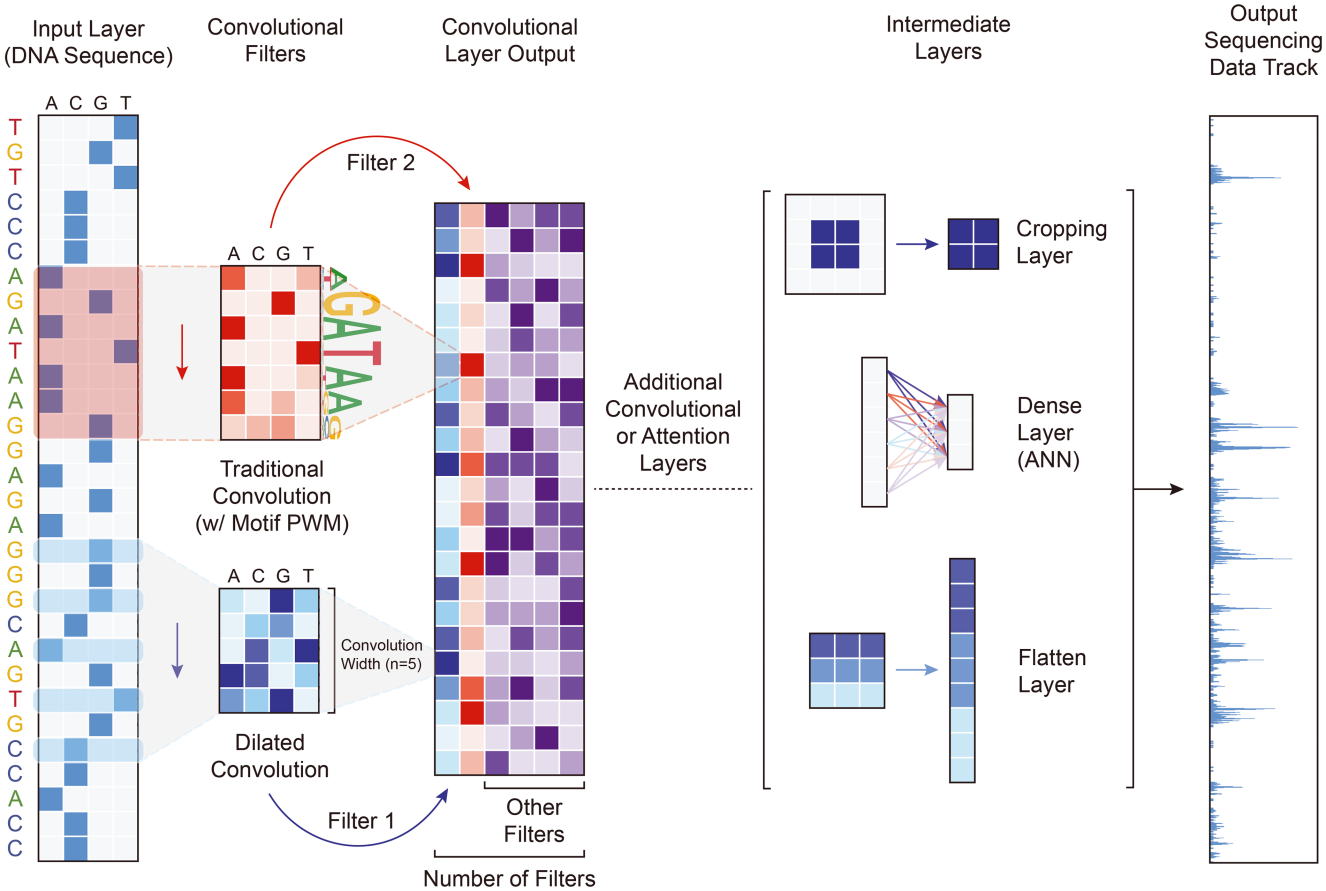
Sample genomics DL model with convolutional, attention, and intermediate layers. This model representation captures the most basic architecture used by most genomics DL models. The input DNA sequence is first one-hot encoded into the 4-by-N matrix shown on the left, then a convolutional layer extracts certain patterns by traversing the input sequence with multiple filters, whose weights are learned during training. Both standard and diluted convolutional layers are shown. Along with more convolutional or attention layers, model designers often use intermediate layers to simplify computation, consolidate data representations, or learn more patterns. Examples of intermediate layers include fully-connected, RNN, cropping, flatten, or pooling layers. Lastly, the model outputs either a predicted genomic track as shown or a single label representing the amount of enriched signal for the entire sequence.

Convolutional neural networks are a type of deep neural network that rely on convolutional filters to traverse an input matrix of values, performing multiplication operations between input values and their corresponding weight followed by a nonlinear transformation (Figure 1B). Originally developed for image processing and classification (Lecun et al., 1998), CNNs have been widely used for applications where it is important to capture local dependencies in spatial or sequential data. In genomic applications, the DNA sequence used as the input to CNN models is commonly represented using one-hot-encoding, which forms a 4-by-N matrix of binary values where N is the length of the DNA sequence and the four rows represent the presence or absence of the bases A, C, G, and T, respectively. This 4-by-N matrix can then be treated as an image and scanned by convolutional filters to extract relevant patterns and features for the target output (Figure 2). These filters consist of a matrix of weights that are each multiplied by the corresponding input element and are often designed in a 4-by-X shape to traverse the DNA sequence like a sliding window. Specialized convolutional layers further optimize model performance, including dilated convolutional layers that expand the convolutional kernel and residual convolutional layers that provide an alternative path to pass information through convolutional layers. Dilated layers increase a convolutional filter’s input context by introducing gaps in the filter elements **(**Figure 2), while residual layers enable much deeper architectures by avoiding the vanishing gradient problem, where back-propagated gradients become extremely small and cannot effectively update model weights in training. In recent years, CNNs have been by far the most popular model architecture used for sequence-based prediction tasks and have been used to model a multitude of biological phenomena and data types including but not limited to general chromatin features (Kelley et al., 2018), TF binding profiles (Avsec et al., 2021b), gene expression (Zhou et al., 2018), 3D chromatin conformation (Fudenberg et al., 2020), DNA methylation (Angermueller et al., 2016), massively parallel reporter assay (MPRA) data (Movva et al., 2019), and RNA splicing (Jaganathan et al., 2019).

Residual Neural Networks (RNNs) are derived from ANNs and designed for tasks with sequential or temporal input data of variable length, having found particular use in natural language processing with problems regarding speech recognition, sentiment analysis, or text translation. RNNs engage in parameter-sharing where each input is passed through the same feedforward neural network architecture, and the output of the previous layer is incorporated into the prediction of the subsequent layer, introducing the idea of short-term memory (Figure 1C; Rumelhart et al., 1986). An extension of the RNN architecture is the long short-term memory (LSTM) unit, which stores salient features in a separate memory state that can encode for temporally extended patterns (Figure 1C; Hochreiter and Schmidhuber, 1997). There are two intuitive advantages of RNNs over CNNs. With short- and long-term memory, RNNs should in theory be able to remember pertinent features and apply them to relevant sequence elements anywhere along a chromosome. Because the identical core model segment is applied to every input element, RNNs are also robust against translational shifts in the indexing of an input DNA sequence. In practice, however, complex CNN architectures have been able to match or outperform RNN models with general sequence modeling (Bai et al., 2018). Moreover, because RNNs rely on sequential operations in training and prediction, they struggle to take advantage of modern GPU parallelization capabilities and are slow to train. Nevertheless, they have found moderate use in genomics, especially with aggregating outputs from convolutional layers (Quang and Xie, 2016; Atak et al., 2021).

Transformers are one of the newest and most powerful model architectures in current literature. First described in 2017, transformer models use multi-head attention layers with self-attention mechanisms that compute global dependencies between every pairwise interaction of input elements (Figure 1D; Vaswani et al., 2017). Unlike convolutional filters or RNN and LSTM memory mechanisms that integrate neighboring input values before more distant elements, these attention weights enable direct information flow between any set of inputs while maintaining distance context through positional encoding, where relative distances are appended into each input’s vector representation. Given such improvements in learning how distant data elements influence and interact with each other, transformers have vast potential for integrating information from much longer input DNA sequences to better capture enhancer-promoter interactions and other gene regulatory patterns. With the increased training parallelization enabled by the attention layers that replace sequential memory, transformer models can be trained faster and with many more parameters than earlier architectures, leading some to argue that transformers are the starting point for another paradigm shift in the field of DL (Bommasani et al., 2022). Transformers have already revolutionized the field of natural language processing (Acheampong et al., 2021) and are quickly gaining traction in genomics research, with multi-head attention layers exhibiting vast performance improvements in modeling chromatin features (Ji et al., 2021) and gene expression (Avsec et al., 2021a; Vaishnav et al., 2022).

### Complex models and transfer learning

In most traditional ML contexts, models are concerned with solving a single prediction task given a single input data type. However, a single-task approach’s performance may be hindered by the lack of available training data that fits the exact purpose. For instance, despite large amounts of genomic data profiling chromatin accessibility or TF binding, this data may come from different approaches (such as ATAC-seq, ChIP-seq, and DNase-seq) and correspond to different cell types. Instead of training a separate single-task model for each data type or cell type instance, multi-task learning can harness the shared features extracted by a single model to jointly predict accessibility scores across multiple outputs (such as genome tracks) (Thung and Wee, 2018). In multi-task learning, it is important to optimize the amount of parameter sharing used by the model, and this is often achieved with a shared ‘trunk’ of convolutional layers for feature extraction followed by individual output ANN branches for each task (Crawshaw, 2020). Multi-task learning has been successfully applied in genomics research to predict chromatin accessibility, gene expression, and TF binding from sequence across data types and cell types (Zhou and Troyanskaya, 2015; Kelley et al., 2016).

Similarly, multimodal models leverage multiple input data modalities to capture a more complete understanding of natural phenomena. This type of architecture first processes each modality using a dedicated submodel then merges the resulting representations through concatenation and relies on a shared model to make the final prediction. Some of the primary challenges for multimodal model research involve data representation and the fusion of modalities before prediction. For an extensive review of these challenges as well as recent advances in multimodal ML, we refer readers to (Baltrušaitis et al., 2019). As an example of a multimodal model, the FactorNet approach concatenated DNase cleavage signal and other optional features such as mapability and gene annotation data as extra rows on the one-hot-encoded DNA sequence to predict cell type-specific TF binding (Quang and Xie, 2019). Another model, PrimateAI, integrated amino acid sequences and position-weight matrix (PWM) conservation scores from both humans and primates to predict the functional effect of missense variants (Sundaram et al., 2018). Data integration strategies for biological data in ML have been reviewed extensively in existing literature (Zitnik et al., 2019).

Transfer learning describes the process by which a new model, due to data scarcity or other limitation, partially reuses another model’s parameters that were trained on a separate but similar domain. Therefore, a model previously trained with a more general purpose and larger available dataset can be adapted to a more specific purpose and refined with a lesser amount of training data due to the inherent shared characteristics between the two tasks (Weiss et al., 2016). One successful implementation in genomics used transfer learning to improve performance of single-task models trained to predict chromatin accessibility in specific cell types by using weights from a separate multitask model (Kelley et al., 2016). Other models use pretrained weights either from existing TF PWMs (Atak et al., 2021) or previously trained models of chromatin accessibility (Schwessinger et al., 2020).

### Model interpretability

On top of making accurate and robust predictions, DL applications in genomics research are used to gain insight into the molecular mechanisms driving a model’s prediction to check for correlation with real-world biology. However, DL model predictions are notoriously challenging to interpret due to the “black box” problem, where the highly-complex weights of a DL model are opaque to human-interpretability because training algorithms update hidden weights and extract salient features without human intervention. In recent years, methods for post-hoc interpretability that enable prediction analysis after training have been developed and popularized to address this issue and are largely classified into perturbation-based methods and backpropagation-based methods. Here, we discuss the model interpretation methods relevant for the models discussed later in this review (Table 1). Interpretability methods for many key models released prior to 2019 have been reviewed previously (Talukder et al., 2021).

**Table 1:** Interpretation methods used by deep learning models of genomic data.

	*In silico* mutagenesis	Kernel analysis	Saliency maps or Gradient × Input	DeepLIFT or DeepSHAP
TF binding and chromatin accessibility	DeepBind (Alipanahi et al., 2015)	DeepBind (Alipanahi et al., 2015)	FactorNet (Quang and Xie, 2019)	BPNet (Avsec et al., 2021b)
	DeepSEA (Zhou and Troyanskaya, 2015)	Basset (Kelley et al., 2016)		ChromBPNet (Trevino et al., 2021)
	Basset (Kelley et al., 2016)	FactorNet (Quang and Xie, 2019)		DeepMEL2 (Atak et al., 2021)
	DeepFIGV (Hoffman et al., 2019)	DeepFIGV (Hoffman et al., 2019)		MPRA-DragoNN (Movva et al., 2019)
	DeepMEL2 (Atak et al., 2021)	DeepFun (Pei et al., 2021)		
		DNABERT (Ji et al., 2021)		
Gene expression	Basenji (Kelley et al., 2018)	Enformer (Avsec et al., 2021a)	Basenji (Kelley et al., 2018)	Xpresso (Agarwal and Shendure, 2020)
	ExPecto (Zhou et al., 2018)		Xpresso (Agarwal and Shendure, 2020)	
	Enformer (Avsec et al., 2021a)		Enformer (Avsec et al., 2021a)	
Chromatin conformation	Akita (Fudenberg et al., 2020)		DeepC (Schwessinger et al., 2020)	
	Orca (Zhou, 2022)			

The most easily applicable and commonly used model interpretation approach for sequence-based DL models is *in silico* mutagenesis (ISM). The name “*in silico* mutagenesis” comes from the initial approach of perturbing bases of a DNA sequence input and observing changes to the model output, such as the level of transcription factor binding predicted from chromatin accessibility data. Using ISM, researchers systematically identify which elements within the input DNA sequence elicit the most significant changes to the model prediction and are thus most “important” to the genomic interactions learned by the model. Basic variant prioritization is often conducted using a simplified version of ISM where individual variant SNPs are perturbed and the change to the prediction is assessed and scored, with higher values interpreted as greater putative functional effects. ISM, as the most straightforward method of model evaluation, was the primary interpretation approach in earlier models (Alipanahi et al., 2015; Zhou and Troyanskaya, 2015; Kelley et al., 2016) and continues to find use in newer models, especially when paired with other more specialized interpretability methods (Avsec et al., 2021a; Zhou, 2022). However, complete ISM is a very computationally expensive process — to assess the effect of every SNP in a N-bp DNA sequence, a model must make 3 N predictions, a value that becomes prohibitively large in the evaluation of long input sequences or many genomic loci.

An alternative simple interpretation method is kernel analysis, where researchers dissect model weights for salient information. Using kernel analysis in CNNs, researchers can investigate the patterns stored in the convolutional filters that traverse the previous layer. Specifically in bioinformatics, CNN kernel analysis is often used to discover sequence motifs that represent biological phenomena such as TF binding patterns. In transformers, kernel analysis can reveal which other regions of the input sequence the model paid the most attention to in making a prediction for the current region. However, as models become more complex and contain a greater number of layers, kernel analysis becomes increasingly obsolete, where sequence motifs or attention interactions may be dispersed across distinct convolutional or attention filters on multiple layers.

To address the drawbacks of ISM and kernel analysis, many backpropagation-based approaches have been developed that more efficiently and accurately interpret model predictions. As described above, backpropagation is the algorithm that updates model weights by measuring the gradient with respect to the loss function, which can be interpreted as a particular input’s importance to the final prediction. Using backpropagated gradients, saliency maps calculate contribution scores for every value in the input matrix, highlighting the input values that can most strongly alter the model’s classification prediction (Simonyan et al., 2014). As an extension to this, the gradient x input method multiplies corresponding elements in the saliency map and input matrix to achieve slightly greater accuracy. ISM only evaluates changes to the final prediction, while kernel analysis only interprets patterns from the model’s internal weights. These two backpropagation-based algorithms incorporate both of those features to capture a more complete picture of the significant input elements within the computed importance scores.

These early backpropagation interpretation approaches struggle with a specific scenario called model saturation. The model saturation phenomenon occurs when the activation of any one or more inputs exhibits a constant output. In this scenario, the gradient for those inputs will be zero in instances where two or more inputs are active, because altering any of those inputs will not affect the output. Therefore, traditional gradient approaches would determine that a change to a single input will negligibly affect the output value and lose this subtle, yet valuable information. More recently, the DeepLIFT algorithm was developed and calculates attribution scores by interpreting predictions within the context of a ‘reference’ value, often dinucleotide-shuffled sequences in genomics (Shrikumar et al., 2019). DeepLIFT avoids the saturation issue by explaining the difference between the actual and reference outputs through the difference between the actual and reference inputs. DeepSHAP builds on the DeepLIFT method by using game-theoretic Shapley values that optimize credit allocation and make local explanations (Lundberg and Lee, 2017). Although these methods have gained some popularity in recent years, they are less straightforward to implement compared to simpler methods and thus will likely see further improvements in the near future.

Instead of input-sequence-specific methods, other interpretability approaches aim to identify global patterns that the model has learned that are not unique to individual test instances. The TF-MoDISco method (Shrikumar et al., 2020) aggregates the outputs of base-resolution contribution scores (from methods such as DeepLIFT and DeepSHAP) into a set of summarized TF motifs that captures regions of heightened importance. Global importance analysis (Koo et al., 2021) fills a similar role and can be used to test the generalizability of user-specified hypotheses against randomized sequence context with controlled ISM methods.

## Genomic applications of deep learning

This section discusses the applications of DL in genomics research, with a particular emphasis on understanding the impact of genetic variants in neurodegenerative diseases. We focus on the key architectural innovations, development directions, and downstream analytical techniques in DL genomics research by first introducing three seminal studies (DeepBind, DeepSEA, and Basset) then highlighting the significant publications from the last 5 years.

As outlined above, noncoding genetic variants represent the vast majority (>90%) of variants associated with diseases through GWASs. As such, understanding and predicting the impacts of noncoding variants on gene regulation and gene expression remains a problem of critical importance to the field of disease genetics. Deep learning models have been widely employed to solve this problem, predicting variant-specific impacts at multiple levels of this complex process. Here, we organize these models into three categories based on the aspect of the gene regulatory landscape that the model is built to predict: (1) transcription factor binding / chromatin accessibility, (2) gene expression output, and (3) 3D chromatin interaction.

Traditionally, DL models in genomics consist of a series of convolutional or multi-head attention layers followed by a series of intermediate layers (including fully connected, RNN, cropping, or flattening layers) that learn the relationship between a one-hot encoded DNA sequence and a region of genomic data **(**Figure 2). Despite predicting different types of information, all of these model categories have been affected by three broader shifts in the trajectory of model development, including the emergence of cell type-specific models, the increase of model input information, and the shift to quantitative rather than categorical model outputs. Perhaps most important to the prediction of activity of noncoding variants, the gene regulatory landscape is highly cell type-specific, implying that models trained to predict aspects of gene regulation should similarly be trained on data derived from well-defined cell types. The human brain is composed of a heterogeneous collection of different cell types, including excitatory neurons, inhibitory neurons, microglia, astrocytes, oligodendrocyte precursors, oligodendrocytes, and endothelial cells. In fact, single-nucleus RNA-seq has been used to define at least 75 distinct classes of brain cells (Hodge et al., 2019). As genetic variants can have divergent effects on different cell types (Nott et al., 2019; Corces et al., 2020), the use of cell type-specific genomic data is an important determinant of a model’s accuracy to real world biology and thus its applicability to disease research.

In addition to accounting for cell type-specificity, model inputs have diversified in two directions, increasing either in input context or resolution. Considerations for the input design vary by purpose, as increasing the input length may, for example, better model the long-range relationships impacting gene regulation and chromosome structure. On the other hand, increasing input resolution may make the model more accurate, interpretable, and robust at a local scale. Early DL models in genomics were trained to predict a singular binary label describing the presence or absence of a peak region in the genomic data. However, given the great importance of continuous data in the interpretation of biological phenomena, more recent DL models of TF binding or gene expression were developed to make quantitative predictions that preserve shape, magnitude, context, and other nuanced features of genomic tracks. These quantitative predictions can be made in different resolutions, with base-resolution models outputting real value predictions at every location on the input sequence and lower resolution models making binned predictions that summarize binding or expression in larger sequence blocks.

### Early implementations

DeepBind (Alipanahi et al., 2015), DeepSEA (Zhou and Troyanskaya, 2015), and Basset (Kelley et al., 2016) were three of the most influential early implementations of CNNs in genomics and all primarily focused on predicting transcription factor (TF) binding. The DeepBind approach demonstrated the applicability of CNN models for predicting binding affinities of TFs and RNA binding proteins (RBPs) using a single convolutional layer as a motif detector. Hundreds of single-task models were trained on one-hot-encoded DNA sequences up to 101 bp in length, enabling DeepBind to outperform existing non-DL algorithms. In DeepSEA, CNNs were trained to predict the presence or absence of TF binding in 919 ChIP-seq or DNase-seq experiments using a 1,000-bp input DNA sequence. The wider input sequence length compared to DeepBind gives the DeepSEA model more information from which to learn, and the three-layered convolutional architecture enables better recognition of complex patterns. DeepSEA has a multi-task architecture, meaning it predicts TF binding simultaneously for all 919 tracks given one input sequence. This model configuration shares the same core layers across distinct predictions and cell types, facilitating better feature extraction and improving performance. With similar features to DeepSEA, the Basset model makes binarized predictions of chromatin accessibility simultaneously in 164 cell types from 600-bp input sequences. Basset’s architecture, constructed with three convolutional layers and two fully connected layers, was one of the first to explicitly leverage cell type-specific data to identify functional variants that may only have an effect in particular cell types. ISM was employed in all three studies to identify important sequence features and nominate functional variants for disease.

All three of these studies implemented kernel analysis methods to study the larger sequence motif patterns learned by the models. Specifically, researchers matched the weights of convolutional filters with TF position weight matrices (PWMs) that represent the sequence patterns with which a TF prefers to bind. The CNN filters from DeepBind and Basset were shown to correspond to known TF motifs, revealing that the models captured genuine biological properties of TF binding in the CNN’s extracted features. However, the patterns discovered through this method become less viable as more layers are added in deeper neural networks because larger genomic patterns may be represented across weights in multiple layers.

To affirm the utility of DL in genomics, these new models must be compared to the previous leading architecture for modeling cell type-specific gene regulatory patterns, the gapped k-mer support vector machine (gkm-SVM) models (Ghandi et al., 2014). SVMs are a subclass of ML algorithms that are trained on support vectors (data points on the boundary of a classification task) to determine the best hyperplane to separate classes in multidimensional space. K-mers are sequence patterns of length k, while gapped k-mers are an optimized version of k-mers that allow for spaces between included nucleotides and better overall modeling of the loose syntax exhibited by TF binding. Given a set of input sequences, gkm-SVM models have been used to accurately distinguish between sequences in peak and nonpeak regions. The deltaSVM method enables interpretation of gkm-SVM models to evaluate the effect that SNPs have on chromatin accessibility and has been used to accurately identify functional variants potentially relevant to disease (Lee et al., 2015).

When benchmarked against gkm-SVM models, DeepSEA and Basset both substantially improved in terms of classification accuracy. As mentioned above, despite this improvement in performance, these first CNN models only output a singular binary or real-value label that collapses the characteristics of a peak into a much less informative and interpretable form. We refer readers to previous reviews (Angermueller et al., 2016; Eraslan et al., 2019; Zou et al., 2019) for more information regarding earlier implementations of DL in genomics.

### Transcription factor binding and chromatin accessibility

The modeling of TF binding and chromatin accessibility continues to be a focal point of DL research in genomics with important applications in understanding disease. The popularization of sequencing technologies such as DNase-seq, ATAC-seq, and ChIP-seq have enabled the genome-wide analysis of gene regulation, and sequence-based DL models have sought to harness the vast amount of generated data to learn the cell type-specific gene regulatory grammar or “cis-regulatory code” that governs the binding of TFs and the activity of regulatory elements. After models have been trained with such genomic data, researchers can predict the effect of a genetic variant by testing both alleles of a SNP separately and identifying any differences in the models prediction based on that single-base change. Large variant effects are presumed to correlate with functional alterations of TF binding, which can theoretically influence gene expression and downstream cellular phenotypes.

As the field matures, approaches for improving model performance and applicability to disease have inevitably diverged. The most straightforward path for further model development has been to refine previous methodologies, and many newer studies built off of the groundwork laid by the three aforementioned early studies. In the context of disease variants, the multitask DeepSEA architecture was extended to investigate the impact of noncoding mutations in autism spectrum disorder (ASD) (Zhou et al., 2019), while an extended version of Basset outperformed the original model and successfully predicted causal variants in the Clinvar archive and the Simons Simplex Collection (SSC) cohort (Pei et al., 2021).

The same research group that produced Basset developed Basenji, an optimized version of the previous architecture that further expanded the input sequence length from 1,000 bp to ~131 kb and makes a ~ 32-kb-long qualitative prediction of the epigenomic profile with a 128-bp resolution rather than a simple binary value (Kelley et al., 2018). This improvement was achieved with dilated convolutional layers that could better capture long-range dependencies, and although the two models cannot be directly compared, Basenji outperformed Basset on binarized chromatin accessibility predictions. Similarly, the authors behind DeepSEA recently published an expanded version of the original multitask model in the form of Sei (Chen et al., 2022), which increased the input context from 1,000 bp to 4 kb and predicts probabilities for 21,907 cis-regulatory profiles instead of 919. With regard to the downstream applications, Sei was used to map sequences into clusters of particular regulatory roles defined as sequence classes. This classification method successfully associated disease mutations with cell type-specific regulatory mechanisms, presenting a more organized and inherently interpretable picture of the gene regulatory landscape than other DL methods.

An alternative philosophy appeared in 2021 with the publication of the BPNet model (Avsec et al., 2021b). Instead of trying to capture more distal elements with a longer input DNA sequence, a CNN was designed to make base-resolution (nucleotide-level) predictions of TF binding. The computational tradeoff of limiting the input DNA sequence allows BPNet to learn the subtle effects on TF binding that can only be captured at a per-base-pair output, such as the precise set of base pairs bound by a given TF. Trained on chromatin immunoprecipitation (ChIP)-nexus data of pluripotency-related TFs in mouse embryonic stem cells, BPNet was used to uncover more nuanced rules of the motif syntax, including the influence of TF binding motifs and their arrangement on TF binding. Using DeepLIFT (Shrikumar et al., 2019), backpropagated importance scores were generated to explain which bases were most important in the model’s prediction. Although this research did not focus on the disease applicability of BPNet, the highly accurate and interpretable modeling of TF binding enabled by BPNet has the potential to be applied to the prioritization of causative variants for diseases such as AD. For instance, the ChromBPNet model was adapted from the BPNet protocol to train with scATAC-seq chromatin accessibility data and used to prioritize functional variants for ASD (Trevino et al., 2021). In this ASD study, DeepSHAP (Lundberg and Lee, 2017), a method similar to DeepLIFT, was used to calculate feature attribution scores for each base in the input DNA sequence and identify the disrupted TF motif responsible for the change in chromatin accessibility.

Cell type-specific predictions have always been important for disease research, as seen in DeepSEA and Basset, and continued research in this direction is crucial for a better understanding of disease etiology in different cell states. To address the issue of data scarcity in cell type-specific TF binding profiles, transfer learning techniques have been employed to incorporate reference cell type chromatin features in training (Quang and Xie, 2019) or augment pre-trained models using gene expression data from a target cell type (Nair et al., 2019). To extract the maximum amount of information from scarce cell type-specific genomic data, instead of just using the reference sequence during training, studies have integrated whole genome sequencing techniques to train models on the actual personalized genetic sequence, exposing the model directly to experimentally accurate variant effects (Hoffman et al., 2019). Combining both of these ideas for disease-specific research, the DeepMEL2 architecture (Atak et al., 2021), which includes a convolutional layer with weights initialized to the JASPAR database’s TF PWMs and a bidirectional LSTM layer, was trained to learn the relationship between whole genomes and matched chromatin and gene expression data in melanoma cell lines. DeepMEL2 outperformed DeepSEA and Basset with robust predictions of functional variant effect in melanocytic samples, confirming the importance of developing “matched” epigenomic models with disease-relevant data to achieve greater predictive power.

As an orthogonal method of predicting regulatory element activity, MPRA-DragoNN (Movva et al., 2019) is trained using massively parallel reporter assay (MPRA) data instead of the more traditional approach using ATAC-seq, DNase-seq, or ChIP-seq. MPRA experiments measure the expression output of candidate cis-regulatory sequences that, with the help of a minimal promoter, control the expression of a barcoded transcript. MPRAs were originally designed to estimate the gene regulatory activity of a given sequence but it has become increasingly common to use these assays to test the differential regulatory activity between reference and variant sequences. This makes MPRAs especially useful tools for variant effect prediction because researchers can test the impact of thousands of SNPs simultaneously *in vitro*. Because the MPRA-DragoNN model is exposed directly to variant sequences during training, the relationships it learns should be more robust and sensitive to variant effects. MPRA-DragoNN is a multi-task CNN architecture with performance on par with the replicate concordance of the MPRA datasets used in training. Model performance was validated using independent MPRA datasets, and the DeepLIFT interpretation algorithm was used to generate importance scores that identified putative TF binding sites. As next-generation sequencing technologies continue to improve and diversify, their incorporation into training pipelines as new input or output data modalities will increase the predictive power and performance of new DL models.

Other researchers have experimented with implementing entirely new model architectures to model TF binding. Transformer models were recently developed for the task of predicting the presence or absence of promoter regions, transcription factor binding sites (TFBSs), and splice sites from short 512-bp sequences (Ji et al., 2021). After tokenizing input sequences into k-mers, the DNABERT model self-supervises a pre-training paradigm that develops attention networks and can then be fine-tuned to specific downstream genomic tasks (such as predicting TF binding, identifying promoter regions, or predicting splice sites), showing promise with functional variant effect prediction.

### Gene expression

Many aspects of cellular state and activity are encoded in the gene expression landscape. By understanding which genes are expressed and at what levels, one can make predictions about what a cell is doing and how it might respond to a given stimulus. Therefore, developing a better fundamental understanding of the mechanisms controlling gene expression can provide key insights into cellular and disease physiology. More specifically, using DL models to study the effects of noncoding variants on gene expression has the power to unveil biological pathways causing cell state disruptions in neurodegenerative diseases. However, gene expression is not solely reliant on the gene’s coding sequence and is still driven by gene regulatory elements that may be hundreds of kilobases away. Thus, while models of TF binding primarily evaluate the ability of TFs to bind at a certain location within the genome, models of gene expression are challenged with incorporating more input information and context to capture the many regulatory interactions associated with a given loci. The rapid adoption of sequencing technologies such as RNA-seq and cap analysis of gene expression (CAGE-seq) has enabled the proliferation of diverse DL applications for predicting gene expression. The optimization of these DL models has required a particular focus on expanding input context, innovating with multi-modal or multi-task architectures, using diverse data representations, and improving distal information flow. Just as previously described with chromatin accessibility models, variant effect prediction can be conducted with gene expression models using sequence perturbations or backpropagation-based interpretation methods.

As a result of the similarities between genomic data for chromatin accessibility and gene expression, some model architectures can be used interchangeably between the two applications. For instance, the Basenji model was also applied to cell type-specific CAGE gene expression profiles, and when interpreted with saliency maps, was able to identify enhancer and promoter regions driving gene expression predictions (Kelley et al., 2018). Basenji2 refined the training paradigm implemented in Basenji by incorporating both human and mouse genomic data in a multi-task framework, improving model performance on unseen and variant sequences (Kelley, 2020). Apart from Basenji, ExPecto (Zhou et al., 2018) is another popular DL approach used for predicting gene expression. The three-part framework of Expecto consists of a CNN extended from the DeepSEA architecture, a spatial transformation module for dimensionality reduction of the CNN’s output features, and a linear regression model to make the final tissue-specific gene expression prediction. Expecto captures a similar input range as Basenji, with 20 kb to either side of the center, successfully identifies causal variants associated with disease, and is interpretable with ISM. Though not outperforming ExPecto, Xpresso (Agarwal and Shendure, 2020) is another CNN-based framework that predicts mRNA abundance using only DNA sequence information after training on only RNA-seq data. Xpresso vastly outperforms other models in its class and is well-suited for the development of accurate cell type-specific models in the context of limited data availability.

The most recent landmark study in DL genomics research came with the publication of the Enformer model (Avsec et al., 2021a), an advanced CNN-transformer architecture used to model sequence-to-expression relationships with the potential to replace the current state-of-the-art CNN models. Enformer’s attention layers capture the importance of every pairwise comparison between the 128-bp resolution bins of the input and enable it to increase information flow between distal regulatory elements up to 100 kb away (compared to CNN model receptive fields of only 20 kb). Whereas convolutional filters and RNN memory rely on neighboring input elements, the multi-head attention layers in Enformer uniquely enable information flow independent of sequence proximity, thus significantly improving feature prioritization of regulatory networks such as enhancer-promoter interactions over long distances. Enformer consistently outperformed state-of-the-art CNN models Basenji2 (Kelley, 2020) and ExPecto (Zhou et al., 2018) with predictions of gene expression, chromatin accessibility, TF binding, and histone modifications as well as variant effect prediction on eQTLs and MPRA data and the interpretation of enhancer-gene association. By leveraging attention layers to enable greater input lengths and information flow, Enformer sets a new standard for DL in genomics. As sequencing data and computational power increase, Enformer-inspired models will only continue to grow. Pairing the Enformer model architecture with potential optimizations for increased resolution, transfer learning for cell type-specific tasks, and 3D chromatin conformation will be key to further improving DL-based variant effect prediction.

### 3D chromatin interactions

While the aforementioned methods try to model the activity of gene regulatory elements or the levels of gene expression using the DNA sequence alone, the genome exists within the 3D space of the nucleus where regulatory elements and target genes that are separated by thousands of base pairs can be brought into close proximity by the formation of chromatin loops. Three-dimensional chromatin conformation plays a critical role in the establishment and maintenance of proper gene expression and thus cell identity. The dysregulation of enhancer-promoter loops can lead to disease-specific alterations in gene expression (Krijger and de Laat, 2016) and has been observed in the context of aging and in AD (Winick-Ng and Rylett, 2018). Specifically, it has been shown that noncoding SNPs can either alter enhancer activity within enhancer-promoter loops (Kikuchi et al., 2019) or the contact frequency of chromatin loops altogether (Greenwald et al., 2019), both impacting downstream gene expression. For instance, the variant rs636317, previously nominated by ML algorithms for variant prioritization (Corces et al., 2020), has been identified as a CTCF binding QTL that disrupts one CTCF anchor of a chromatin loop and upregulates the MS4A6A gene implicated in AD (Novikova et al., 2021b). More broadly, the disruption of CTCF binding and chromatin conformation by alteration of the CTCF binding motif has been widely observed in cancer (Katainen et al., 2015; Guo et al., 2018; Liu et al., 2019), suggesting that similar mechanisms may be at play for non-somatic variants affecting other diseases. Moreover, the recent discovery of the MGMT risk gene for AD in women was made possible through the use of Hi-C data which enabled the discovery of relevant SNPs and DNA methylation sites (Chung et al., 2022). Many DL approaches have been used in recent years to predict 3D genome interactions from DNA sequence with CNN or CNN-LSTM architectures, including enhancer-promoter relationships (Singh et al., 2019), CTCF/cohesin insulator loops (Trieu et al., 2020), and probabilities of chromatin interaction between pairs of open chromatin regions (Cao et al., 2021). However, these models rely only on local sequence regions and lose broader chromosome context. A new style of DL framework has emerged that instead predicts the qualitative contact map that represents degrees of 3D chromatin interaction between genome regions of a continuous sequence collected by Hi-C and micro-C experiments. This approach appears to have a greater applicability to disease genomics research because chromatin interactions and effects can be analyzed and perturbed in the actual genome location and context.

The Akita model (Fudenberg et al., 2020) consists of a two-part architecture to learn the relationship between a DNA sequence and Hi-C or Micro-C contact frequency maps. A “trunk” derived from the Basenji CNN architecture first processes a 1-Mb input sequence into a profile of 2048-bp bins. Then, a “head” block transforms those 1D representations into a 2D matrix that is passed through 2D convolutional layers and maps to a matrix of its 3D contact points. Using ISM, the authors were able to verify the importance of CTCF and other motifs for 3D chromatin conformation and predict the effects of genetic variants on 3D chromatin interactions.

The authors of DeepC (Schwessinger et al., 2020) harnessed the power of transfer learning by first training a CNN on chromatin accessibility data and attaching those same convolutional weights to more dilated convolutional and fully connected layers to predict chromatin interactions from Hi-C data. In this study, the pairwise interaction Hi-C data were encoded as matrices where the columns were 5-kb bins and the rows were distances from the given bin up to 1 Mb. Using saliency maps, the authors of this study similarly found that CTCF sites and active promoters were most important for the model’s prediction.

Most recently, the Orca framework (Zhou, 2022) proposed a new CNN-based architecture with a multi-resolution encoder and cascading decoder that makes predictions at nine resolutions ranging from 4 to 1,024 kb on window sizes from 1 to 256 Mb. This unique architecture captures an input range of up to 256 Mb (more than the largest human chromosome), which enables multi-resolution predictions that capture various features from chromatin compartment formation at the chromosome scale to topologically associating domains at the sub-1-Mb scale to TF binding motifs at the nucleotide scale. Orca successfully reproduced variant effect predictions of structural variants that agreed with experimental results and nominated CTCF and cell type-specific TF motifs as primary contributors to local structural remodeling (<1 Mb). It also significantly outperformed Akita on the Micro-C dataset that was used to train both models. From this diverse set of DL methods modeling TF binding, gene expression, and chromatin conformation, neurodegenerative disease researchers can effectively model the genomic landscape of disease-implicated cell types and evaluate GWAS risk loci to nominate candidate causal variants for AD.

### Noncoding variant prediction

Although these DL models are not necessarily optimized for the task of variant effect prediction, post-hoc interpretability methods are used to derive variant annotations and impact scores. To validate that the candidate causal SNPs nominated by these DL models accurately represent real variant effects and are suitable for the task at hand, model predictions are compared with a form of “ground truth” generally derived from sequencing or other genomic data. The most common method of assessing the real-world compatibility of DL predictions is by testing a model’s ability to reproduce quantitative trait loci (QTLs), which are statistical representations of differential activity between the two alleles of a variant at specific locations within the genome. This approach is observed in many of the aforementioned studies, including Enformer, Expecto, Basenji, DeepMEL2, Akita, Sei, and DeepSEA just to name a few. Strong prediction accuracies observed with respect to QTLs demonstrate the capabilities of these DL approaches to indirectly but accurately model functional variant effects. Alternatively, researchers can also compare their model’s predictions against MPRA data that directly test sequence perturbation effects *in vitro*, as seen in Enformer, Expecto, and MPRA-DragoNN. However, as MPRAs do not test the effects of variants within the correct genomic context, the use of MPRA data as a ground truth may not be ideal. Given the high accuracies achieved through these validation methods, it has become clear that DL models of genomic relationships are highly capable of understanding differential regulatory activity between alternate and reference sequences in genetic variants.

## Machine learning in neurodegenerative disease genomics research

This section discusses the implementation of machine learning techniques within neurodegenerative disease research. We first give a brief background on clinical ML implementations, which dominate the vast majority of AD-related ML research. Next, we discuss the current studies at the intersection of ML, genomics, and AD research that are applicable to variant effect prediction and prioritization, a domain still in its infancy. Although we primarily center our discussion around AD, we also demonstrate that the same methodologies are transferrable to other neurodegenerative disease contexts such as Parkinson’s disease or ALS. Lastly, we elaborate on the current state of AD genomics research, with a particular focus on using the available data to study neurodegeneration using the aforementioned general DL genomics methods.

In current neurodegeneration research, ML has been primarily used for clinical applications, namely the stratification of patients and the development of new treatments. DL approaches have been used to facilitate AD diagnosis using inputs consisting of (i) patient gene expression and DNA methylation data (Park et al., 2020), (ii) blood gene expression data (Lee and Lee, 2020), (iii) MRI neuroimaging data, clinical test results, and genetic SNP profiles (Venugopalan et al., 2021), and (iv) clinical, demographic, and MRI data (Hashemifar et al., 2022). Many of these ML studies in AD attempt to distinguish between disease cases and controls and are trained using data derived from the Alzheimer’s Disease Neuroimaging Initiative (Mueller et al., 2005) or the publicly available data sets (Clough and Barrett, 2016). Apart from earlier AD diagnosis, ML has also been used to identify biomarkers and causative variants that increase risk of disease (Jo et al., 2022), classify potential AD risk genes (Huang et al., 2021), and nominate existing therapeutics that could potentially be repurposed for AD given a list of disease-relevant molecular mechanisms (Rodriguez et al., 2021). However, our focus in this review remains on the use of ML/DL methods to predict the functional effects of genetic variants. We refer readers to a recent review (Myszczynska et al., 2020) for a more extensive coverage of clinical-focused applications of ML in AD research.

Sequence-based ML genomics techniques applicable to variant effect prediction are slowly being adopted into the field of neurodegeneration and AD research. In a recent study that sought to profile the epigenomic landscape of brain cell types for Alzheimer’s and Parkinson’s disease research, gkm-SVM machine learning classifiers were trained on each of the 24 single-nucleus ATAC-seq clusters to distinguish between transposase-accessible and inaccessible chromatin (Corces et al., 2020). When used to predict the functional effects of candidate Alzheimer’s and Parkinson’s disease SNPs in GWAS loci that had passed a tiered multi-omic analysis process, the gkm-SVM classifiers were able to nominate new functional noncoding variants and associated genes for further investigation. GkmExplain importance scores were used to interpret model predictions and discover motifs responsible for the allelic impact of the SNPs. Replacing the gkm-SVMs with more complex and historically more accurate DL models may unlock greater predictive power for the discovery of functional disease variants. Nevertheless, variants identified by this study (such as rs636317, rs6733839, and rs13025717) and their associated molecular pathways have been further analyzed and functionally validated using myeloid QTL data (Novikova et al., 2021b), CRISPR/Cas9-mediated deletion of an enhancer region (Nott et al., 2019), and MPRAs (Cooper et al., 2022). This biological validation of ML-nominated SNPs demonstrates the value of ML as a powerful method for variant prioritization with real-world applicability.

Many fine-mapping studies have also used DL models as part of their scoring paradigm to rank candidate GWAS variants by their likelihood of causing neurodegenerative disease. In a meta-analysis of noncoding variants from recent AD GWASs, DeepSEA and SpliceAI prediction scores of differential TF binding and splicing were incorporated as one of many metrics for variant prioritization (Schwartzentruber et al., 2021). DeepSEA scores were influential in the identification of many top variants nominated by this study, such as rs268120 in the SPRED2 locus, rs6733839 in the BIN1 locus, rs7920721 in the ECHDC3 locus, and rs1870137 in the TSPAN14 locus, making determinations of gene regulatory changes such as decreased DNase 1 hypersensitivity or reduced TF binding of USF, HNF4, FOXA1, and SP1 factors. The SpliceAI DL model (Jaganathan et al., 2019) was also used to detect splicing changes and nominated rs4311 in the ACE locus and rs4147918 in the ABCA7 locus. A similar fine-mapping study on Parkinson’s disease GWAS loci (Schilder and Raj, 2022) used the DeepSEA, Basenji, and IMPACT (Amariuta et al., 2019) DL models to functionally annotate consensus SNPs. For instance, Basenji and IMPACT functional effect scores were able to differentiate between rs11088398 and rs2835757 at the DYRK1A locus and determine the causal variant, supporting the observations from enhancer-promoter and peak analysis methods. Another recent study that sought to identify risk variants in amyotrophic lateral sclerosis (ALS) used a CNN model trained on annotated epigenetic features (DHS mapping data, histone modifications, target gene functions, and TF binding sites) to prioritize variants by their functional effect (Yousefian-Jazi et al., 2020). The CNN models were used to prioritize over 8 million SNPs from an ALS rare variants dataset and identified 1,326 putative risk variants for disease. The subsequent fine-mapping analysis nominated two noncoding variants along with their gene targets. The variant rs2370964 was a positively predicted CNN SNP that disrupts CTCF binding in the enhancer site of CX3CL1, a gene associated with microglial toxicity in ALS. Another CNN-nominated variant, rs3093720, decreases the expression of the TNFAIP1 gene and affects the NR3C1 TF associated with heightened stress signaling in neurodegeneration.

These early implementations demonstrate the increasing importance of DL methods for variant prioritization in neurodegenerative disease research (Novikova et al., 2021a). Working orthogonal to the statistical methods and traditional analytical methods for noncoding variant prioritization, DL has the potential to assist the variant fine-mapping process on a large scale, particularly with the influx of new risk loci from recently conducted GWASs.

More specifically, although the field of neurodegeneration research still largely relies on non-ML techniques to analyze genomics data, the eventual integration of DL methods will be crucial for studying the effect of noncoding variants without being hindered by resource or time limitations of biological experiments. Large consortia aimed at mapping out the transcriptional and gene regulatory landscapes of the human genome have generated vast amounts of sequencing data for brain cell types implicated in neurodegenerative diseases. These consortia projects include the ENCODE Project (Moore et al., 2020), the Roadmap Epigenomics Consortium (Kundaje et al., 2015), the Human Cell Atlas (Regev et al., 2017), the Human BioMolecular Atlas Program (Snyder et al., 2019), the Allen Brain Atlas (Sunkin et al., 2013), and Tabula Sapiens (The Tabula Sapiens Consortium, 2022), just to name a few. At the same time, recent AD GWASs continue to discover new genetic loci associated with the disease (de Rojas et al., 2021; Wightman et al., 2021; Bellenguez et al., 2022), showing the continued need for accurate methods for variant effect prediction and candidate variant prioritization. All of the lessons learned from the disease-independent DL genomics methods developed in the last few years can be applied to AD-specific data and use cases. By training those models on relevant cell type-specific sequencing data, researchers can implement appropriate downstream model interpretation algorithms and conduct functional variant effect prediction to identify candidate causal variants from a larger list of GWAS variants and nominate potential biological pathways for neurodegenerative diseases. This paradigm has recently been applied to many other diseases and conditions, including autism spectrum disorder (Trevino et al., 2021), coronary artery disease (Turner et al., 2022), congenital heart disease (Ameen et al., 2022), and ocular diseases (Wang et al., 2022). All of these studies successfully used sequence-based ML models to model genomic characteristics and prioritize functional noncoding variants, indicating that similar methods have strong potential in the field of neurodegeneration.

We believe that as the field continues to grow, it is important to dedicate efforts toward not just clinical ML implementations that focus on diagnosing patients and developing therapeutics but also a more fundamental understanding of the genetic and epigenetic drivers of neurodegenerative diseases. We reiterate that DL models are uniquely positioned to synthesize patterns and salient information from the growing amounts of next-generation sequencing data to conduct variant analysis on the new risk loci nominated by GWASs. The continued development of new optimized model architectures, data processing techniques, and model interpretability methods will likely improve our ability to investigate the genetic basis of AD and associated dementias from the perspective of noncoding variation and give us a better understanding of AD pathogenesis.

## Current limitations and future directions

Challenges associated with DL in disease genomics can be split between data science and model engineering. Within the data science category, genomic data scarcity and data partitioning techniques remain the most pertinent obstacles. Next generation sequencing technologies simply have not been around long enough to collect sufficient data for all relevant cell types and genomic features. However, with the proliferation of large consortia projects, it seems likely that data scarcity issues will be resolved within the next decade. Of course, new genomic technologies will continue to be developed and the state-of-the-art will continue to evolve but we believe that the current wave of single-cell and multi-omic focused data generation will provide an ideal foundation on which to build and train highly effective DL models. In the meantime, transfer, multi-task, and multimodal learning approaches have shown the greatest potential to address data scarcity as addressed above. On the other hand, data preprocessing and partitioning are notoriously challenging with complex genomic data, and those issues are unlikely to be resolved with the advent of new sequencing technologies. The technical logic behind many of these difficulties, which lies outside the scope of this review, is explained in a guide on the common pitfalls with using ML in genomics (Whalen et al., 2022).

As model architectures and development methods continue to diversify, sustained growth in the field will require efficient frameworks for model creation, exchange, and comparison. For example, DL tools can assist genomic research by directing researchers to functional variants with which more specialized experiments can be designed, saving both time and resources. To bridge the gap between disease genomics and DL research, bioinformaticians need tools to either (1) develop DL models themselves or (2) use pre-trained DL models to investigate biological phenomena such as the effect of genetic variants. For the first scenario, the AMBER framework is a fully-automated platform that trains CNNs to learn genomic regulatory features from sequence with accuracies on par with existing expert-designed models (Zhang et al., 2021). AMBER’s integrated downstream applications include prioritization of functional genetic variants and determination of disease enrichment, highly useful functions for the discovery of disease variants. For the second scenario, the Kipoi repository fills the need for a model sharing platform (Avsec et al., 2019). At the time of this publication, Kipoi contains over 2,200 models in 35 model types that users can easily access through a common API and apply to downstream tasks such as variant effect prediction and prediction interpretation. The standardized approach to using models in Kipoi holds great potential for comparing model performance, training new models through transfer learning, and developing compound models that sequentially incorporate predictive models of elementary biological phenomena (i.e., combining models of TF binding and 3D chromatin conformation). The sheer number of different model designs can quickly cloud what actually improves model performance in a specialized scenario and what development paths are worth exploring. To address this confusion, researchers developed the GOPHER model evaluation framework that provides a paradigm to isolate drivers of performance improvements (Toneyan et al., 2022). GOPHER can benchmark the predictive performance of binary and quantitative models, assess the robustness of predictions to noise and minor input perturbations, compare interpretability protocols, and evaluate a model’s applicability to functional variant effect prediction.

## Discussion

The current state-of-the-art DL variant effect prediction pipelines have relied on GWASs to nominate an initial superset of candidate SNPs. From there, variants are scored and prioritized by DL algorithms and associated interpretation methods that nominate a subset of SNPs that are predicted to have a functional effect. This functional effect ranking paradigm varies on a case-by-case basis but generally derives a score from the predicted difference between both alleles of the variant. While successful, these methods have two major areas for improvement.

First, GWASs generally do not have the statistical power to identify rare or structural variants that may have equally important implications for disease (Auer and Lettre, 2015). For example, in AD, rare variants and structural variants account for an equal share of disease susceptibility as common variants (Ridge et al., 2013). Rare variants, defined arbitrarily as those variants occurring in less than 1% of a population, often have stronger effect sizes than common variants (Bomba et al., 2017), making research in this direction critical. Recent AD family-based studies have identified novel rare variants associated with AD (Prokopenko et al., 2021), but such statistical methods are limited to similar family-based studies where increased statistical power can be obtained with a comparatively smaller number of individuals. Expanding such analyses to the broader AD population will require hundreds of thousands of whole genomes accompanied by in depth clinical and neuropathological assessments. In the interim, DL variant effect prediction methods are well-suited for prioritizing rare variants for downstream functional validation. Of course, without the statistical association of a variant with a disease, the primary challenge with functionalizing rare variants will be proving that they play an important role in disease pathogenesis. However, given the clear understudied role that rare variants play in disease susceptibility, we view this as an ideal application of DL with a ripe future in identifying novel disease biology.

Critically, after DL methods are used to refine a list of potentially impactful variants, downstream functional validation of model predictions must be conducted to verify variant effects in real biological contexts. Such functional validation has taken many forms in the literature which range in the level of confidence they provide for the specific variant effects. Because noncoding gene regulatory elements are highly context- and cell type-specific, we argue that it is imperative to perform functional validation in as close to the correct *in vivo* context as possible. However, the lack of strong cross-species conservation of most noncoding regions (Yue et al., 2014) makes it nearly impossible to perform such validation experiments using rodent models of AD. Moreover, while DL methods are able to prioritize large numbers of variants, we are still often left with more variants than could be tested one-by-one. To this end, high-throughput MPRA experiments can be used to test both alleles of a variant in a relevant *in vitro* cell type context and can be applied to thousands of variants in a single experiment (Mulvey et al., 2021). Such MPRAs provide an orthogonal method for functional interpretation of noncoding variant effects. However, these assays are performed outside of the correct genomic context, either episomally or *via* random lentiviral integration, making such assays an imperfect but important proxy for understanding the effects of noncoding variants. With current technologies, perhaps the most rigorous and convincing validation of variant effects comes from scarless single-base editing, whereby a single base is genetically engineered to create a fully isogenic system that can be used to test downstream changes in gene regulation, gene expression, and cellular phenotypes caused by a specific variant. When performed in induced pluripotent stem cells which can be differentiated into many different disease-relevant cell types, such genetic engineering experiments can identify the sufficiency of noncoding variants in the correct genomic context. While DL holds great promise to nominate disease-relevant genetic variants, variant validation in realistic biological contexts is a crucial step before any genomics-derived research can arrive at clinical applications and a necessary process to resolve questions surrounding the disease applicability of DL models.

As genomic DL approaches all attempt to model slightly different aspects of one larger human biological system, combining models represents an attractive approach for defining the genomic features driving disease pathogenesis. For instance, the effect of a noncoding variant can first be predicted with a model of TF binding and then traced through models of 3D genome organization and gene expression to uncover the enhancer-promoter interactions and the associated downstream gene that might mediate the disease-relevant phenotype caused by the variant. Modeling complex biological phenomena in smaller steps and linking models post-development has the potential to greatly increase current model utility to disease research.

In the next decade, we envision that the field of neurodegeneration research will find it important to bridge the gap between genomics research and clinical treatments. At a time when neurodegenerative diseases still lack effective therapies, we believe that pinpointing genomic pathways and targets driving disease phenotypes will be crucial for accurate patient prognostication, sub-selection of patients most likely to respond to specific treatments or clinical trials, and the development of novel therapeutic approaches. With the advent of greater computational power and new algorithmic developments alongside the growth of next generation sequencing technologies, deep learning has the potential to revolutionize disease genomics research, not only with variant effect prediction but also broadly with predicting patient outcomes and prioritizing high potential clinical trials.

## Author contributions

AL wrote the review with guidance and revision by MC. All authors contributed to the article and approved the submitted version.

## Funding

This work was supported by NIH R00-AG059918, U01-AG072573, P01-AG073082, UM1-HG012076 to MC and a gift from the Ray and Dagmar Dolby Family Fund (to the Gladstone Institutes). MC was additionally supported by the Farmer Family Foundation Parkinson’s Research Initiative.

## Conflict of interest

The authors declare that the research was conducted in the absence of any commercial or financial relationships that could be construed as a potential conflict of interest.

## Publisher’s note

All claims expressed in this article are solely those of the authors and do not necessarily represent those of their affiliated organizations, or those of the publisher, the editors and the reviewers. Any product that may be evaluated in this article, or claim that may be made by its manufacturer, is not guaranteed or endorsed by the publisher.
